# Comparison of foveal avascular zone between optical coherence tomography angiography and fluorescein angiography in patients with retinal vein occlusion

**DOI:** 10.1371/journal.pone.0217849

**Published:** 2019-06-04

**Authors:** Jens Ulrich Werner, Felix Böhm, Gabriele E. Lang, Jens Dreyhaupt, Gerhard K. Lang, Christian Enders

**Affiliations:** 1 Department of Ophthalmology, Ulm University, Ulm, Germany; 2 Ulm University, Ulm, Germany; 3 Institute of Epidemiology and Medical Biometry, Ulm University, Ulm, Germany; Boston Medical Center, Boston University School of Medicine, UNITED STATES

## Abstract

**Objective:**

To compare area of foveal avascular zone (FAZ) in different retinal vascular layers in optical coherence tomography angiography (OCTA) and fluorescein angiography (FA) in patients with retinal vein occlusion (RVO).

**Design and methods:**

Prospective cross-sectional comparative study in 47 eyes of 47 patients. FA was recorded with the Zeiss FF450plusIR camera and OCTA was obtained with the Zeiss Cirrus 5000 equipped with the AngioPlex module. Area of FAZ was graded by two independent investigators and calculated with Adobe Photoshop. Analysis for the total study population as well as subgroup analysis for branch retinal vein occlusion (BRVO), central retinal vein occlusion (CRVO) and patients with and without macular edema (ME) was performed.

**Results:**

For all patients, FAZ was 0.449 mm^2^ in FA, 0.496 mm^2^ in OCTA superficial capillary layer (SCL) and 3.168 in OCTA deep capillary layer (DCL). In patients without ME FAZ was 0.288 mm^2^ in FA, 0.342 mm^2^ in OCTA SCL and 1.384 mm^2^ in OCTA DCL. FAZ area measurement in patients with ME revealed 0.482 mm^2^ in FA, 0.527 mm^2^ in OCTA SCL and 3.554 mm^2^ in OCTA DCL.

**Conclusions:**

Especially the SCL of OCTA shows a good agreement to FA in measurement of FAZ in all patients with low limits of variation in patients without ME. There were no considerable differences in BRVO and CRVO. OCTA could replace FA in FAZ area measurement in patients with RVO, especially in those without ME, achieving similar measurements whilst being non-invasive

## Introduction

Retinal vein occlusion (RVO) is a potentially blinding disease with a prevalence of about 0.5%. It is the second most common vascular retinal disease after diabetic retinopathy [1]. Although RVO can be diagnosed by clinical examination alone in most cases, fluorescein angiography (FA) is used to visualize the foveal avascular zone (FAZ), the perfusion situation of the retina and to detect macular edema (ME). It is therefore at present the gold standard in diagnosis and therapy planning.

Visual prognosis depends on the amount of retinal ischemia and ME [2]. It has also been shown that the diameter of the FAZ is inversely correlated with the best corrected visual acuity (BCVA) [3, 4]. RVO can be categorized in ischemic and nonischemic vein occlusion depending on the area of non-perfusion in FA. It has been shown that ischemic central retinal vein occlusion (CRVO) is associated with a poor prognosis regarding visual acuity (VA) and a higher risk for secondary complications like neovascular glaucoma compared to nonischemic CRVO [5].

FA has been the gold standard in RVO diagnosis for many decades. It is well established but the incidence of adverse reactions is high, at 10%. One of 200.000 FA may result in a patients death [6]. Hence the search for a non-invasive technique which at the same achieves at least non-inferior diagnostic accuracy. Optical coherence tomography angiography (OCTA) is a novel diagnostic option for non-invasive assessment of microperfusion in both retina and choroid. The Zeiss Cirrus 5000 is equipped with a line scan ophthalmoscope and operates at 68.000 A-scans per second. Moving erythrocytes cause a contrast between two short repeated B-scans from the same location and so act as an intrinsic contrast medium. This difference in contrast is displayed as perfusion of vessels. The Fast-Trac Eye-Tracking-system can compensate minimal eye movements and improves image quality. This technique enables a depth-selective view of blood flow in different retinal layers: The Zeiss Cirrus 5000 with Angioplex module separates the superficial capillary layer (SCL; from inner limiting membrane to inner plexiform layer), deep capillary layer (DCL; from inner plexiform layer to outer plexiform layer) and avascular layer (from outer plexiform layer to inner segment/outer segment junction of photoreceptors) [7]. Thus OCTA has the potential of becoming the new gold standard in RVO diagnostics.

The aim of this study was to compare the size of the FAZ in FA vs SCL and DCL in OCTA.

## Patients/ Materials and methods

47 eyes of 47 patients with RVO consecutively seen in our tertiary care hospital between 01/2016 and 12/2016 were included in this prospective cross-sectional comparative study. Inclusion criteria were patients with the clinical diagnosis of RVO and validation in FA that involved the macula. Exclusion criteria were severe opacifications of optical media (minimal required signal quality: 5/10), other retinal diseases that affects evaluation of FA or OCTA and uncontrolled glaucoma. Patients unable to hold their head on the chinrest during examination were also excluded.

The study was approved by the institutional review board (Institutional review board of the University of Ulm, application number 388/15) and was conducted in accordance with the declaration of Helsinki. All patients gave their written informed consent to study participation.

### Ophthalmologic examination

A medical history of general and eye disease was conducted. BCVA testing was followed by a slit lamp examination and indirect ophthalmoscopy with dilated pupil. Fundus photography, optical coherence tomography (OCT), FA and OCTA were performed.

### Fundus photography and fluorescein angiography

**Fig 1:**
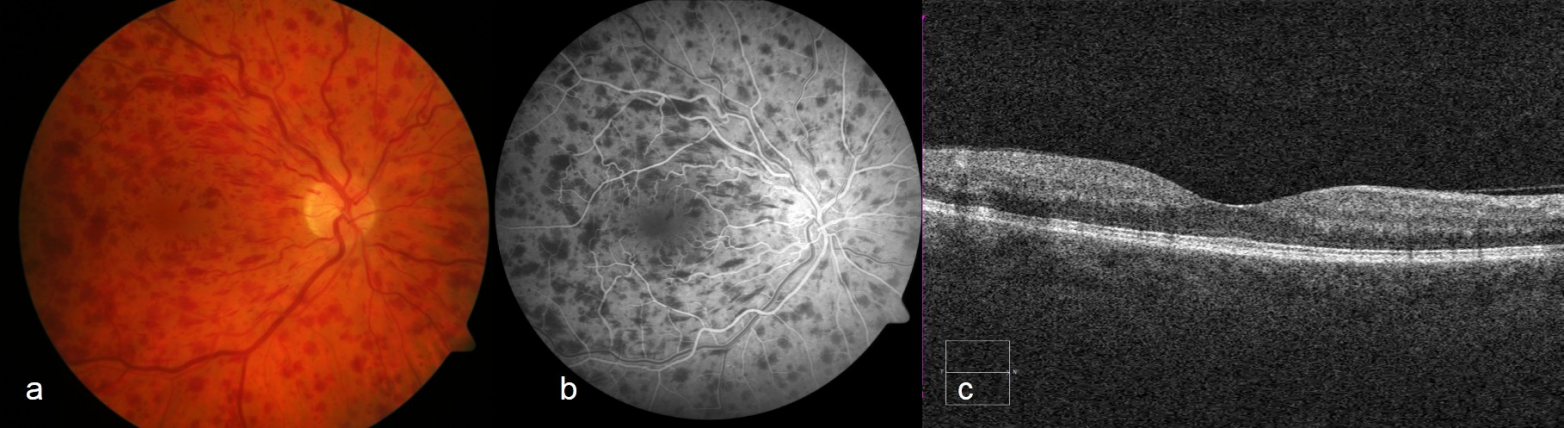
Patient example with CRVO. Fundus photography of the right eye with intraretinal hemorrhages (a). FA 34 s after dye injection showing blockage of retinal and choroidal fluorescence by intraretinal hemorrhages (b). Structural OCT reveals no cystoid macular edema (c).

Fundus photography (Fig 1A) and FA was performed with a Zeiss FF450plusIR camera (Carl Zeiss Meditec, Inc., Dublin, USA). All patients received a 5 ml injection of 20% sodium fluorescein (Fluorescein Alcon 10% Injektionslösung, Alcon, Fort Worth, Texas, USA). Photographs were taken 15–40 seconds after injection (Fig 1B).

### OCT and OCTA

OCT examination was performed with the Carl Zeiss Meditec Inc. Cirrus 5000 equipped with the AngioPlex module, Software Version 9.5.1.13585 (Carl Zeiss Meditec, Inc., Dublin, USA). A structural macular cube was obtained (Fig 1C) as well as an OCTA. Analyzed segmentations in OCTA were superficial retina and deep retina slabs according to the segmentation algorithm of Zeiss. A 3 x 3 mm square was used to obtain highest resolution because of the higher scan densitiy.

### Evaluation of OCTA and FA

**Fig 2:**
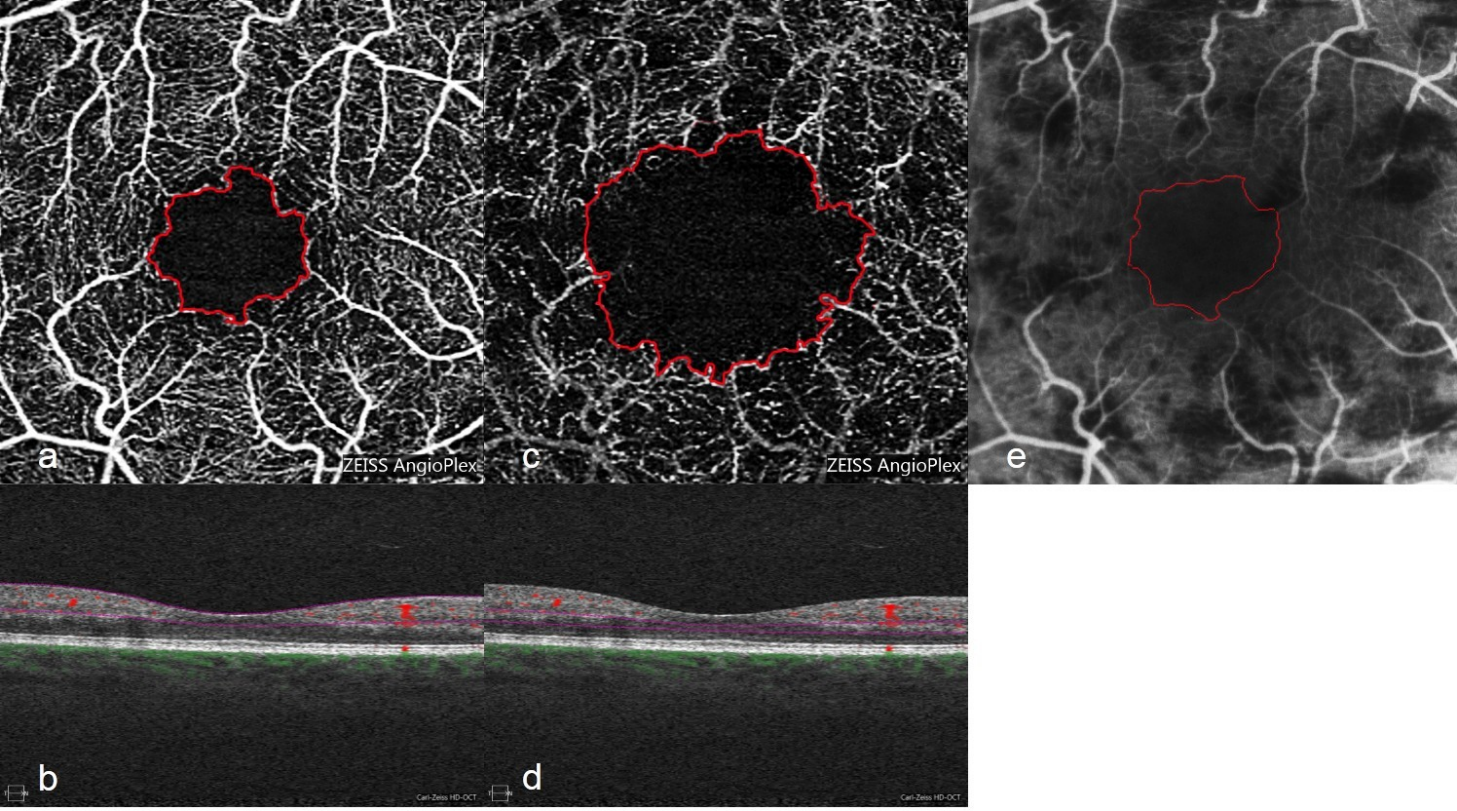
Prepared OCTA and FA images for evaluation of FAZ. Same patient as shown in Fig 1. OCTA SCL with red bordered FAZ (a). Segmentation is displayed below (b). OCTA DCL with red bordered FAZ (c) with corresponding segmentation in (d). FA with red bordered FAZ (e).

Recordings of FA and OCTA (SCL (Fig 2A and 2B) and DCL (Fig 2C and 2D)) were imported to Adobe Photoshop (Adobe Photoshop CC 2018 (19.0), Adobe Systems, San José, California, USA) as BMP-files. FA recording was overlaid to the OCTA SCL as well as the DCL recording; size and picture detail of FA was adjusted to that of OCTA recording (Fig 2E). Two readers (FB and JUW) marked FAZ independently. In case of different markings a third reference reader (GEL) defined the borders of FAZ. Area quantification was also performed in Adobe Photoshop.

### Statistical analysis

The agreement between FA and OCTA (SCL and DCL) was investigated using Bland-Altman-Plots (comparison of the magnitude of the difference vs. the mean of both methods). Analysis was performed for all patients. A subgroup analysis was done for patients with and without ME.

Because of the explorative nature of this study, no adjustment for multiple testing was made. The results of all statistical tests are interpreted in an exploratory sense and not a proof of efficacy. Statistical analyses were conducted using SAS, version 9.4 (SAS Institute Inc, Cary NC, USA).

## Results

A total of 47 eyes from 47 consecutive patients with RVO were included. Table 1 summarizes the patient demographics.

**Table 1:** Patient demographics.

total	47 eyes/47patients
male/female	24/23
mean age (years); standard deviation	66.9 ± 13.0
right eye/left eye	21/26
BRVO/CRVO	33/14
mean BCVA	0.342
macular edema	39/47 (83%)

**Fig 3:**
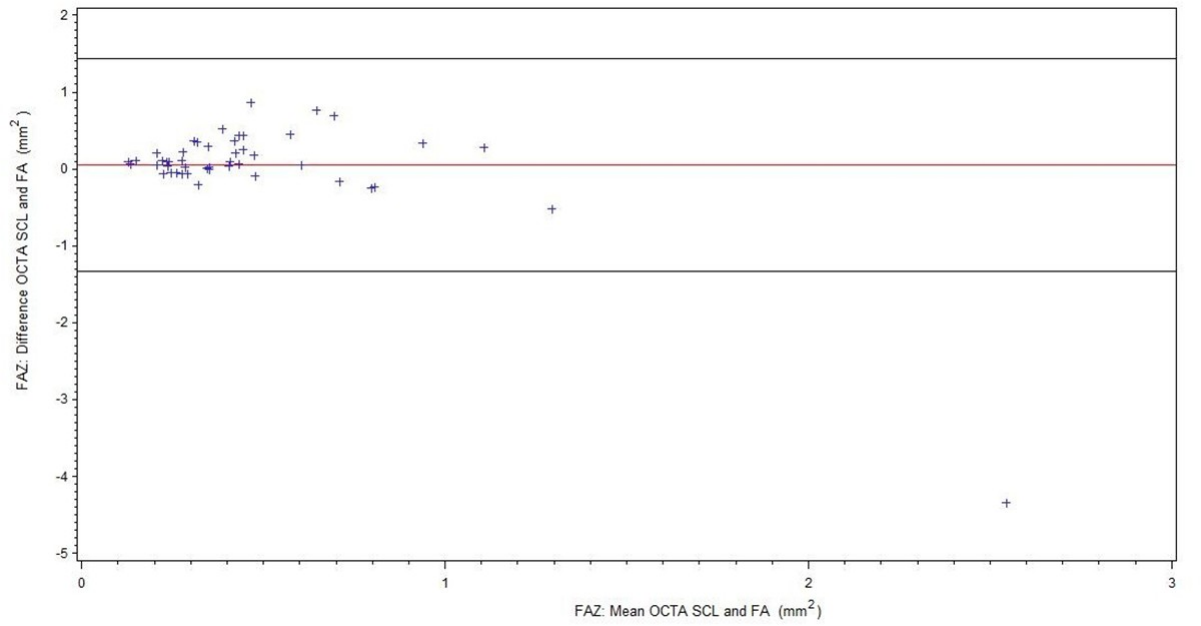
FAZ: Bland-Altman-Plot for the agreement of FA and OCTA SCL for all patients.

**Fig 4:**
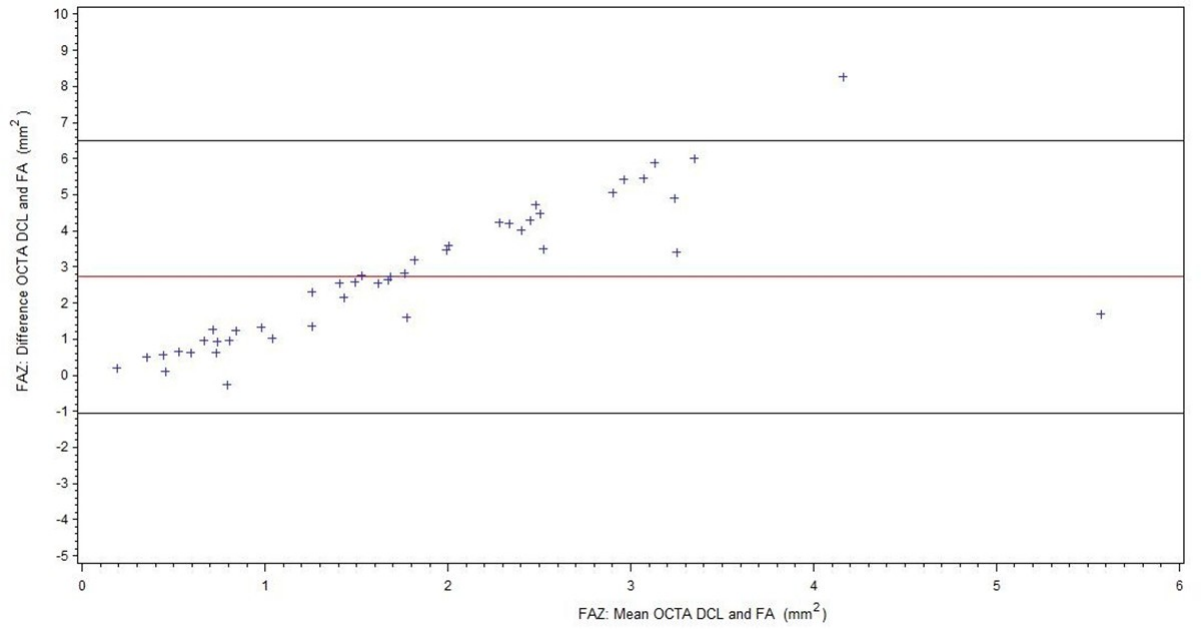
FAZ: Bland-Altman-Plot for the agreement of FA and OCTA DCL for all patients.

**Fig 5:**
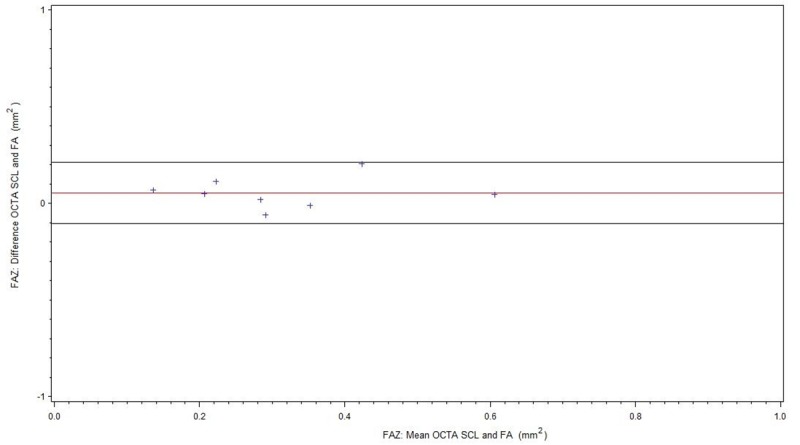
FAZ: Bland-Altman-Plot for the agreement of FA and OCTA SCL in patients without ME.

**Fig 6:**
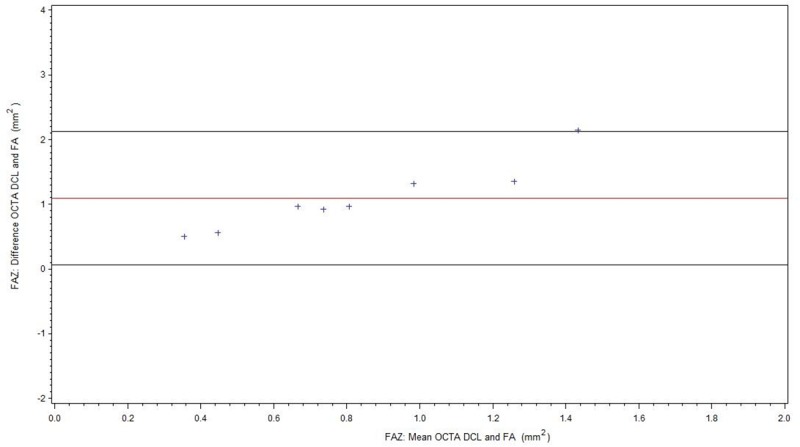
FAZ: Bland-Altman-Plot for the agreement of FA and OCTA DCL in patients without ME.

**Fig 7:**
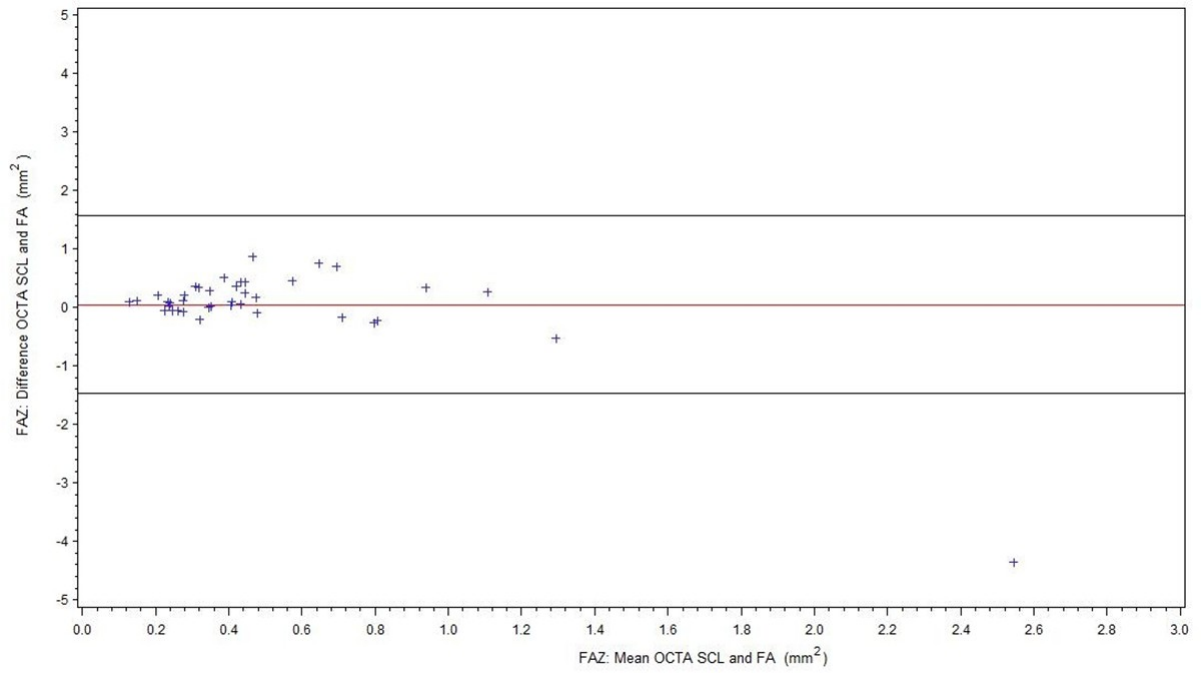
FAZ: Bland-Altman-Plot for the agreement of FA and OCTA SCL in patients with ME.

**Fig 8:**
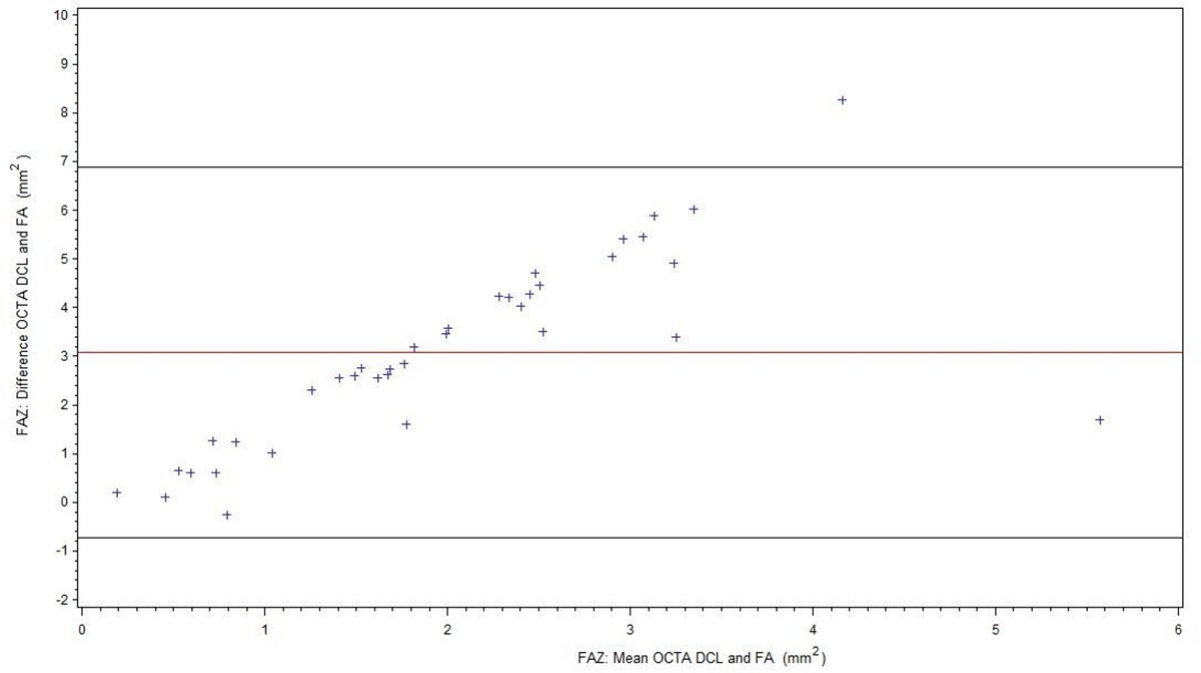
FAZ: Bland-Altman-Plot for the agreement of FA and OCTA DCL in patients with ME.

For readability, all results of the FAZ measurements are summarized in Table 2. Fig 3 shows comparison of FA and OCTA SCL for all patients (BRVO/CRVO), Fig 4 of FA and OCTA DCL. The subgroup without ME is displayed in Fig 5 (FA/OCTA SCL) and Fig 6 (FA/OCTA DCL). Fig 7 (FA/OCTA SCL) and Fig 8 (FA/OCTA DCL) show the subgroup with ME.

**Table 2:** FAZ in FA and OCTA (SCL and DCL) for all patients and the subgroups CRVO/BRVO, with and without ME, is shown in the left column (mean values). The middle column reflects the mean difference between FA and OCTA (SCL and DCL). Limit of agreement between FA and OCTA (SCL and DCL) is shown in the right column.

	FAZ (mm^2^)			Mean difference OCTA–FA (mm^2^)		Limit of agreement FA with OCTA (mm^2^)	
	FA	OCTA		OCTA		OCTA	
		SCL	DCL	SCL	DCL	SCL	DCL
BRVO + CRVO wtih and without ME	0.449	0.496	3.168	0.046	2.724	+1.431 to -1.338	+6.495 to -1.046
without ME	0.288	0.342	1.384	0.054	1.095	+0.212 to -0.104	+2.124 to -0.067
with ME	0.482	0.527	3.554	0.045	3.076	+1.566 to -1.477	+6.873 to -0.720
BRVO with and without ME	0.487	0.483	2.831	-0.004	2.350	+1.599 to -1.608	+5.817 to -1,118
without ME	0.244	0,270	1.268	0.026	1.023	+0.160 to -0.107	+2.326 to -0.280
with ME	0.530	0.5207	3.131	-0.010	2.605	+1.735 to -1.755	+6.147 to -0.938
CRVO with and without ME	0.360	0.526	3.914	0.166	3.554	+0.772 to -0.439	+7.582 to -0,474
without ME	0.362	0.462	1.577	0.100	1.215	+0.279 to -0.079	+1.631 to +0.799
with ME	0.360	0.544	4.552	0.184	4.192	+0.865 to -0.497	+7.802 to +0.581

## Discussion

The mean age of patients with RVO, gender-neutral distribution and the higher prevalence of BRVO than CRVO in our patient cohort is comparable to epidemiological data [8], which let us assume that our study population was representative.

FA is considered the current gold standard in FAZ area measurement. Non-invasive techniques of measurement like OCTA are preferable but need to demonstrate at least non-inferiority to FA. Therefore OCTA needs to achieve a very high level ofimage quality. Ho et al. have shown that scan size selection in OCTA has a clear impact on FAZ size measurement: The 3x3 mm scan delineates FAZ size more clearly than the 6 x 6 mm scan. This is due to the higher scan density and thus higher resolution in the 3x3 mm scan as compared to 6x6 mm and 8x8 mm volume scans [9]. Therefore we chose the 3 x 3 mm scan protocol. In most cases delineation of FAZ was easier in OCTA SCL than in FA.

In our study, FAZ measured in patients with BRVO and CRVO by FA was larger in patients with ME compared to patients without ME. This is remarkable as masking effects due to capillary leakage that induces ME could cause an underestimation of FAZ. This finding is supported by the results of the OCTA SCL measurement that also shows a larger FAZ in patients with ME than in patients without ME. This seems plausible as it has been shown that the breakdown of the blood-retina barrier causes ME [10] and is triggered amongst others by ischemia [11]. OCTA DCL also showed a larger FAZ in patients with ME compared to patients without ME. However, we believe this to be an artefactual finding as discussed below.

FAZ size measurement in SCL of OCTA was a little bit larger compared to FA but shows a good agreement. This result is in accordance with previous findings [12, 13] and holds true for patients with and without ME. However, in patients with ME the limit of agreement is greater than in patients without ME.

In contrast there was a poorer agreement in DCL analysis compared to FA. DCL tends to obtain larger FAZ size measurements than in comparison to SCL of OCTA or FA. This was more pronounced in patients with ME than in patients without ME. Therefore, agreement between FA and OCTA DCL was not present for FAZ. Vessel compression and displacement of perfused tissue out of the segmentation lines due to the edema could be the cause. This indicates that the FAZ which has been measured for many decades using FA is represented by the SCL or the sum of SCL and DCL. Our results are in accordance to those obtained by Coscas et al. who concluded that the FAZ in FA represents mainly the SCL [14]. The Bland-Altman plots for the FAZ in DCL showed that FA measurements of FAZ size were comparable to those obtained by OCTA where the FAZ areas was small. However, this agreement did not exist in larger FAZ: the larger the FAZ, the larger the difference with higher values for the DCL. The relationship between the difference in FAZ measured between the two methods and FAZ size appeared to be linear. As this relationship was observed in both subgroups, in patients with and without ME, it is implausible segmentation artifacts are to blame. It is possible that in patients with severe RVO the DCL is more affected than the SCL.

As far as we know this is the first study that compares FAZ size measurement in FA with SCl and DCL of OCTA in patients with RVO with a subgroup analysis of BRVO, CRVO, patients with and without macular edema. Furthermore this study is one of the largest that analyzes FAZ in RVO patients with OCTA. Results for patients with BRVO and CRVO are comparable. The limit of agreement for FA and OCTA SCL is in a some smaller range for patients with CRVO and ME than for patients with BRVO and ME. However the mean difference OCTA SCL-FA is slightly better for the BRVO subgroup so that there is no clear disadvantage of using OCTA in patients either with BRVO or CRVO.

In summary, analysis of OCTA SCL can replace FA in FAZ area measurement in patients with BRVO and CRVO, especially in those without ME. A next step could be the comparison of the area of nonperfusion in FA and OCTA using the automontage feature now available on widefield OCTA devices as indicated by Kimura et al. who concluded, that widefield imaging with OCTA is useful in evaluation of retinal ischemia [15]. Here, the pace of technological development for both OCTA hardware and software seems promising.
